# Death from Meningeal Apoplexy

**Published:** 1870-05

**Authors:** James S. Bailey

**Affiliations:** Albany, N. Y.


ART III.—*Death from Meningeal Apoplexy.* BY JAMES S. BAILEY, M. D. *Albany, N. Y.*

The busy practitioner meets daily, in nervous females, cases denominated hysteria; and so many peculiar phases do they assume, that even the most experienced observer is often in doubt as to the true diagnosis.

The necessity of great caution in making and expressing an opinion, should be deeply impressed upon the young practitioner by a history of the following case, which was in many particulars obscure until death speedily solved the mystery.

Mrs. F., aged thirty-six, was the mother of four children; the youngest, nine years old. Her catamenia appeared August 22d; on the morning of August 23d, she was awakened by a singular sensation within the cranium. She complained of headache and dimness of vision, and soon after became insensible. In the course of half an hour I saw her. She was then in a semi-conscious state, but would answer any question in the affirmative; was very restless, and would not take anything unless forced down her. Pulse normal, and extremities cool. There was no change in the pupil of the eye, and no paralysis. Six hours later she was seized with a hard convulsion, after which, bloody foamy mucus exuded from her mouth, and her breathing seemed slightly interrupted.

This continued throughout the day, as did her unconsciousness. The next morning she swallowed readily anything given her, but seemed bewildered. She now complained of intolerable headache, and could not sleep. This condition lasted about one week, and gradually subsided, followed by a stiffness of her neck, and extreme pain in her back and extremities, which continued three or four days, in spite of efforts to relieve her. She then rested well, her appetite returned, and her symptoms indicated speedy recovery. She expressed herself as feeling quite well except debilitated. Upon the morning of the 7th of September, about the same hour as first attacked, she awakened the family by making a peculiar noise. Her condition was then found to be much the same as when first attacked, with the exception of slight paralysis on the right side. There was no stertor. By 9 o'clock, A. M., she was again seized with a hard convulsion and expired.

Two years prior she miscarried, had dangerous hemorrhage, followed by puerperal peritonitis. After this she was always anæmic. Her urine was not albuminous.

Autopsy, seven hours after death—body spare but not emaciated; skin dusky. Head—there was a large clot in the arachnoid cavity, covering the convolutions lying under the left parietal and temporal bones; smaller one on the corresponding part of the right hemisphere. Base of brain and sheath of spinal cord enveloped in a thick clot of blood. The left ventricle was filled with a clot which had partly escaped into the right ventricle, through the foramen of Monro. The fourth and fifth ventricles were also filled with clots. All of these clots appeared fresh, as if recently effused. At the lower part of the left hemisphere in the cerebral tissue was another isolated clot, which was partially discolored, and the brain substance around it was stained yellow and somewhat softened. Thorax—lungs and heart normal. Abdomen—liver large and elongated, spleen and pancreas normal. Both kidneys were very large, covered with cysts, some of which contained a straw colored and others a dark grumous fluid. The left kidney which was rather the larger, measured 11 inches in length by 4 inches in width.* Uterus normal in size and appearance. Os tincae rather strawberry-like. Ovaries large and a fresh *corpus luteum* found in the left one.

* We have received a stereoscopic view of the kidney described, and would furnish a cut of it for our journal but for the impossibility of conveying any better idea of it than has been done verbally by Dr. Bailey. Both the case and the pathological specimen are of great interest; every case thus carefully observed and fully reported, is a positive addition to the literature of such disease.—Ed.

